# Association between reduced muscle mass and poor prognosis of biliary sepsis

**DOI:** 10.1038/s41598-024-52502-9

**Published:** 2024-01-22

**Authors:** Soh Yeon Chun, Young Soon Cho, Han Bit Kim

**Affiliations:** 1 Department of Emergency Medicine, Soonchunhyang University Gumi Hospital, Gumi, Republic of Korea; 2 Department of Emergency Medicine, Soonchunhyang University Bucheon Hospital, 170, Jomaru-ro, Bucheon-si, Gyeonggi-do Republic of Korea

**Keywords:** Infection, Infectious diseases, Medical research, Risk factors

## Abstract

Sepsis is a life-threatening disease, contributing to significant morbidity and mortality. This study aimed to investigate the association between low muscle mass and the prognosis of patients with biliary sepsis, focusing on outcomes such as length of hospital stay (LOS), intensive care unit (ICU) admission, and in-hospital mortality. This retrospective, single-center, observational study included adult patients with biliary sepsis who visited the emergency department between January 2016 and December 2021. Low muscle mass was assessed using the psoas muscle index (PMI). Using computed tomography imaging, the area of both sides of the psoas muscle at the L3 level was measured, and the PMI, corrected by the patient’s height was calculated. The primary outcome was in-hospital mortality, and the secondary outcomes were intensive care unit (ICU) admission, LOS, and 14-day mortality. A total of 745 patients were included in this study. Low muscle mass was defined as a PMI < 421 mm^2^/m^2^ for males and < 268 mm^2^/m^2^ for females with the lower quartile of PMI according to sex. The cohort was classified into sarcopenic (n = 189) and non-sarcopenic (n = 556) groups. There was a significant association between low muscle mass and in-hospital mortality (odds ratio, 3.81; 95% confidence interval, 1.08–13.47; p < 0.001), while there was no significant association between low muscle mass and ICU admission. In addition, the median LOS in the sarcopenic group (10 [7–14] days) was significantly longer than the median (8 [6–11] days) in the non-sarcopenic group. Low muscle mass was significantly associated with clinical outcomes, particularly in-hospital mortality and LOS, in patients with biliary sepsis.

## Introduction

Sarcopenia, i.e. muscle failure, is a muscle disease rooted in adverse muscle changes that accrue across a lifetime; sarcopenia is common among adults of older age but can also occur earlier in life. Sarcopenia is defined by low levels of measures for three parameters: (1) muscle strength, (2) muscle quantity/quality and (3) physical performance as an indicator of severity^1^. While aging is the primary factor associated with sarcopenia, other factors such as chronic inflammatory conditions (e.g., malignant tumors, cardiovascular and pulmonary disease, and inflammatory conditions) can also contribute to its development^2,3^. Acute sarcopenia can also occur in acute inflammatory conditions, such as sepsis, leading to rapid muscle loss^4^. The concept of acute sarcopenia has emerged recently from research on chronic sarcopenia, defined as sarcopenia that evolves in less than 6 months, and is usually triggered by an acute stressor^5^. Although research on muscle mass loss in acute severe illness is limited compared to that in chronic conditions, studies suggest that skeletal muscle plays a crucial role in physical recovery, rehabilitation, and long-term functionality after hospitalization^6^.

Various methods are available to measure muscle mass and strength loss. To measure muscle strength, one such method is gripping strength, which is measured using a calibrated portable dynamometer. Accurate measurement involves interpreting the data based on population norms^7^. Another method is the chair stand test, which evaluates leg muscle strength and endurance by measuring the time taken to perform five consecutive chair stands without using the arms^6,8,9^. To measure muscle mass, anthropometry is occasionally used to reflect nutritional status in the geriatric patients but is not suitable for precise muscle mass measurement. However, calf circumference measurement can serve as a diagnostic tool when other methods are not available^10^. Calf circumference measurements have limitations because they cannot be generalized to other ethnic groups or elderly people with frail health conditions^11^.

Using CT or MRI is one of good options to measure muscle mass loss. However, the measurement of all muscles in the body using CT or MRI is limited. Previous studies have investigated whether specific muscles proportionally reflect overall muscle mass^12–14^.

Shen et al. reported a linear relationship (R^2^ = 0.86) between the total skeletal muscle area from whole-body MRI and the skeletal muscle mass from a 5 cm CT cross-sectional image above the L4-5 intervertebral disc^13^. They also found a linear relationship between psoas muscle cross-sectional area on CT and whole-body skeletal muscle mass^12,13^.

Sepsis affects nearly 50 million people worldwide annually and contributes to over 11 million deaths, despite access to high-quality medical services^15^. Sepsis, severe sepsis, and septic shock are common conditions seen in emergency rooms and ICUs, with mortality rates ranging between 30 and 60% despite appropriate treatment^16–18^. The prognosis of patients with sepsis is significantly influenced by early recognition and the prompt initiation of appropriate therapy^19^. Therefore, early recognition and appropriate treatment in the emergency room can significantly affect patient prognosis^20^.

Physiological scoring systems, such as the Sequential Organ Failure Assessment (SOFA) score, are commonly used to evaluate sepsis severity and predict prognosis. However, these scoring systems focus solely on the physiological status and do not consider frailty in geriatric patients. Incorporating frailty assessment alongside physiological status may improve prognostic accuracy^21–24^. Sarcopenia and physical frailty were associated and partly overlap, especially on parameters of impaired physical function^25^. Studies have explored the relationship between sarcopenia and the prognosis of sepsis^6^. However, considering sepsis as a single disease is challenging, because its prognosis varies depending on the underlying cause^26^. Hence, our study aimed to investigate the association between low muscle mass and prognosis in a specific disease with a uniform prognosis. A recent study analyzed sarcopenia and prognosis in acute cholecystitis-induced sepsis^27^ However, there is no research on biliary sepsis, severe disease due to its course and its significant association with relevant diseases, either benign or malignant, of the biliary tract, pancreas, hepatic hilus^28^. Therefore, our study aimed to explore the association between low muscle mass and the prognosis of biliary sepsis.

## Material and methods

### Study design and population

This retrospective, observational study was conducted at a tertiary university hospital in Bucheon, South Korea. The study protocol was approved by the Institutional Review Board of Soonchunhyang University Bucheon Hospital (IRB file no. 2022-12-010). Due to the retrospective nature of the study, the need of informed consent was waived by Institutional Review Board of Soonchunhyang University Bucheon Hospital. This study had been performed in accordance with the Declaration of Helsinki. This study included patients presented emergency department between January 2016 and December 2021. Patients with biliary sepsis who visited the emergency department during the study period were included. Sepsis was identified when a patient exhibited a Sequential Organ Failure Assessment (SOFA) score of 2 or more, accompanied by clinical indication of infection^27^. Patients under 18 years of age, those who were discharged or died within 24 h, those who did not undergo CT, those with a Do Not Resuscitate (DNR) status, those with Discharge Against Medical Advice (DAMA), those transferred to another hospital, and those with other final diagnoses were excluded^27^. The conclusive diagnosis was ascertained through a combination of medical documentation and the radiologist’s analysis of the CT findings^27^. Sepsis due to biliary cause and other diseases were classified accordingly.

### Variables

Data including age, sex, medical comorbidities, and initial vital signs were collected from the medical records of the enrolled patients. Initial laboratory test results, including arterial blood gas, complete blood count, and serum chemistry, were also collected. Records of procedures or surgeries performed to treat the cause of biliary sepsis and their clinical outcomes were documented. To determine the severity of sepsis, the SOFA and Quick Sequential Organ Failure Assessment (qSOFA) scores were calculated^27^. The SOFA and qSOFA score were assessed based on the evaluation of patients’ clinical findings and diagnostic test results at the time of their emergency department presentation. The primary outcome was in-hospital mortality, and secondary outcomes included intensive care unit (ICU) admission, length of hospital stay (LOS), and 14-day mortality^27^.

### Measurement on CT imaging

**Figure 1:**
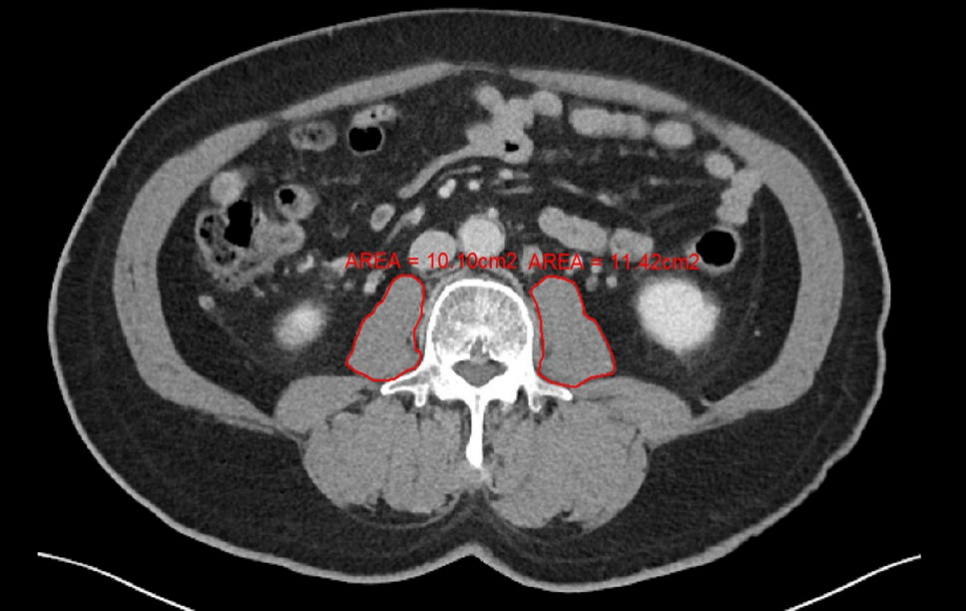
Measurement of cross-sectional area of psoas muscle at L3 vertebra level.

Abdominal CT scan images were used to assess the psoas muscle area. Consistent with previous studies, cross-sectional images at the level of the L3 transverse process were selected^29–32^. Researchers, who were blinded to patient information, traced the psoas muscle margins using an area assessment software to calculate the total psoas area (TPA) (Fig. 1)^27^. The TPA was normalized to the individual’s height to obtain the psoas muscle index (PMI): PMI = Total psoas muscle area at the L3 level/height^2^ (mm^2^/m^2^)^27^.

### Statistical analysis

All statistical analyses were performed using the R software version 4.0.3 for Windows (R Foundation for Statistical Computing, Vienna, Austria). Utilizing the established PMI threshold, patients were categorized into either the sarcopenic or non-sarcopenic cohorts. Categorical variables are denoted by counts and proportions, while continuous ones that don't follow a normal distribution are indicated by median values and their interquartile ranges (IQR). Kolmogorov–Smirnov test was used to evaluate the distribution of continuous variables. To contrast these variables, the study used the Chi-square or Fisher's exact test and the Wilcoxon rank-sum test, adopting a significance criterion of p < 0.05. To discern factors independently influencing clinical results, logistic regression was used. Variables deemed significant for the logistic regression were chosen through a univariate analysis, which assessed the association between clinical outcomes and independent factors. However, not all factors, which found to be statistically significant in univariate analysis, were included in the multivariate analysis. Factors which were commonly used in previous studies for sepsis prognosis and sarcopenia were selectively included. To evaluate the performance of logistic model, Brier score was calculated. To evaluate the predictive power of the logistic model against SOFA and qSOFA scores, Receiver Operating Characteristic (ROC) curves were drawn.

## Results

**Figure 2:**
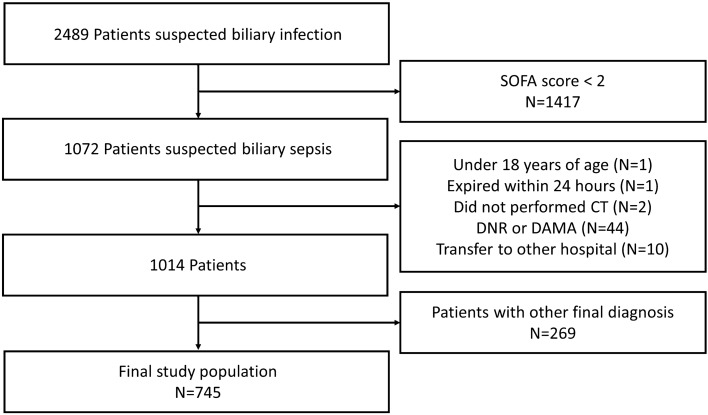
Flow chart of patient enrollment in this study.

Between January 2016 and December 2021, 2489 patients with biliary sepsis presented to the ED. Of this cohort, patients with SOFA score less than 2 (n = 1417) were precluded from the study. After screening the remaining 1072 patients, the following exclusions were made: those below 18 years of age (n = 1), fatalities within the first 24 h (n = 1), patients who had undergone CT (n = 2), patients with directives of DNR or DAMA (n = 44), inter-hospital transfer (n = 10), and those with differential final diagnoses (n = 269). Consequently, 745 patients were deemed eligible and included in the final analysis (Fig. 2).

**Figure 3:**
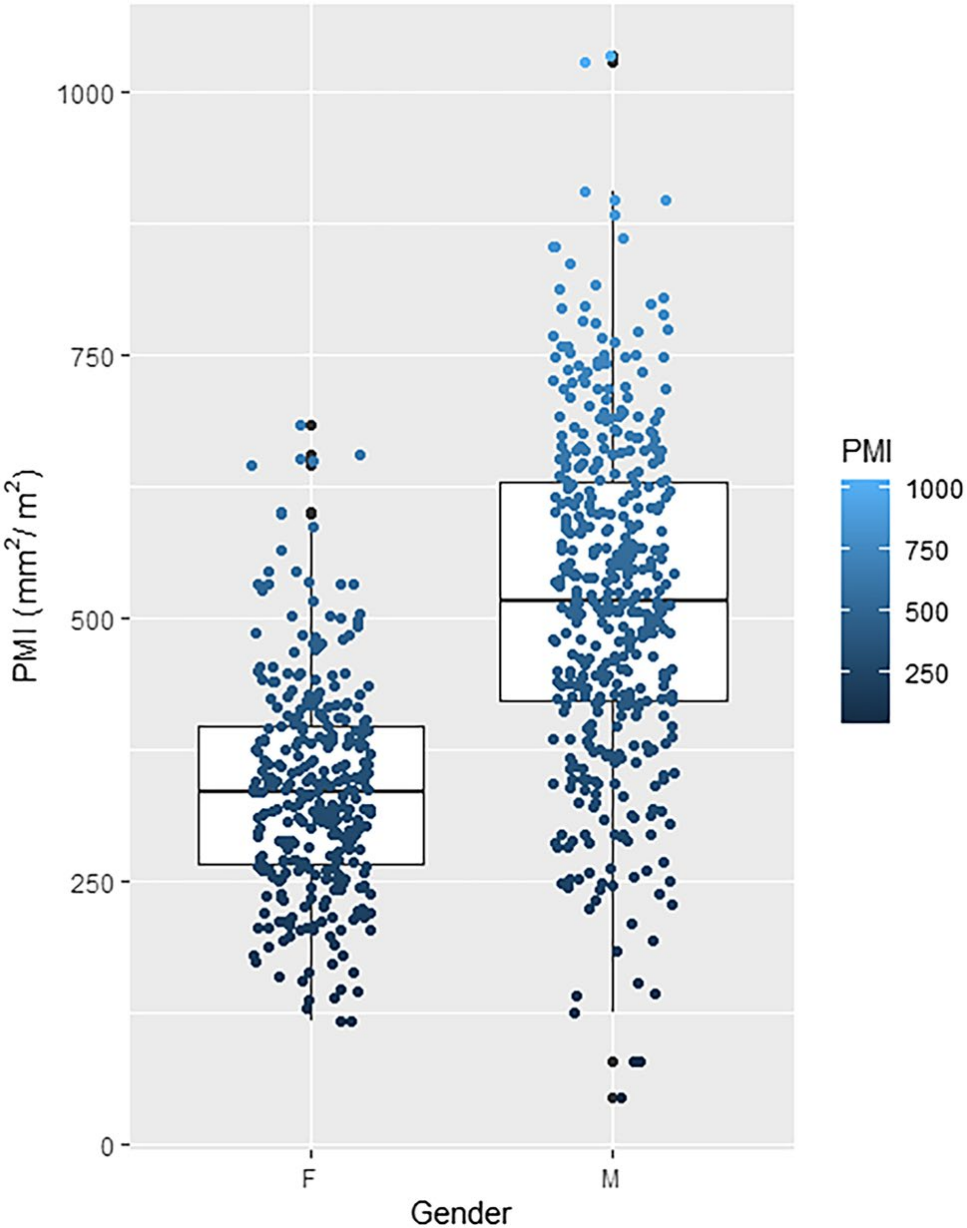
Boxplot showing the distribution of PMI by gender in this study cohort.

In previous studies, if the muscle area values of the cohort were normally distributed, the mean minus two standard deviations (mean-2SD) was used as the cut-off value for low muscle mass^33–39^. If the muscle area values were not normally distributed, the lowest quartile was used as the cut-off value. In this cohort, cut-off values for corresponding to the lowest quartile were set as PMI < 421 mm^2^/m^2^ for men and < 268 mm^2^/m^2^ for women (Fig. 3). Patients were classified into a non-sarcopenic group (n = 556) and a sarcopenic group (n = 189). The baseline characteristics of each group are shown in Table 1. The sarcopenic group was significantly older than the non-sarcopenic group (median 77.0 vs. median 68.0 years) and had a significantly lower median body mass index (median 21.2 vs. median 24.3 kg/m^2^). When comparing non-sarcopenic and sarcopenic groups, the proportion of comorbidities, including diabetes, heart failure, liver cirrhosis, and malignant neoplasia, was significantly higher in the sarcopenic group. In terms of initial vital signs, the sarcopenic group had significantly lower median mean arterial pressure (MAP) (median 93.3 vs. 96.7 mmHg) and O_2_ saturation (96.0 vs. 97.0%). In laboratory tests, the sarcopenic group had significantly higher levels of blood urea nitrogen (BUN) (20.6 vs. 16.2 mg/dL), C-reactive protein (CRP) (8.1 vs. 4.7 mg/dL), and lactate (2.7 vs. 1.8 mg/dL), but significantly lower levels of albumin (3.6 vs. 4.0 g/dL). Further, the sarcopenic group had a significantly higher SOFA score (median 3, IQR 2–5 vs. 3, 2–4). The proportion of qSOFA scores in the sarcopenic group was less than 2 points in 88.9% of patients and 2 points or more in 20.1%, whereas in the non-sarcopenic group, it was 95.8% and 4.2%, respectively.

**Table 1:** Comparison of general demographics and clinical variables between sarcopenic and non-sarcopenic patients.

Variables	Non-Sarcopenic (N = 556)	Sarcopenic (N = 189)	P
Age, years (median, IQR)	68.0 (55.0–78.0)	77.0 (66.0–83.0)	< 0.001
Gender, male	318 (57.2%)	107 (56.6%)	0.957
BMI, kg/m^2^ (median, IQR)	24.3 (22.3–26.8)	21.2 (19.2–23.7)	< 0.001
Comorbidities (N, %)			
Hypertension	270 (48.7)	101 (53.7)	0.273
Diabetes mellitus	157 (28.2)	71 (37.6)	0.021
Heart failure	20 (3.6)	17 (9.0)	0.006
COPD	13 (2.3)	4 (2.1)	1.000
Liver cirrhosis	15 (2.7)	12 (6.3)	0.037
CKD	56 (10.1)	18 (9.6)	0.950
CVA	60 (10.8)	30 (15.9)	0.085
Neoplasm	62 (11.2)	39 (20.6)	0.002
Initial vital signs (median, IQR)			
SBP, mmHg	130.0 (116.5–150.0)	120.0 (105.0–140.0)	< 0.001
DBP, mmHg	80.0 (70.0–90.0)	80.0 (64.0–90.0)	0.039
MAP, mmHg	96.7 (86.7–110.0)	93.3 (80.0–106.7)	0.003
HR, beats/min	89.0 (77.0–100.0)	92.0 (80.0–107.0)	0.037
RR, breaths/mm	20.0 (18.0–20.0)	20.0 (20.0–20.0)	0.187
BT, °C	37.2 (36.7–38.0)	37.3 (36.6–38.2)	0.610
O_2_ saturation, %	97.0 (95.0–98.0)	96.0 (94.0–98.0)	< 0.001
Laboratory findings (median, IQR)			
WBC, *1000	10.3 (7.5–14.5)	11.7 (8.0–16.0)	0.017
Platelets, *1000	186.5 (136.0–239.5)	165.0 (124.0–229.0)	0.044
Albumin, g/dL	4.0 (3.6–4.3)	3.6 (3.1–4.0)	< 0.001
BUN, mg/dL	16.2 (11.9–21.8)	20.6 (13.6–31.5)	< 0.001
Creatinine, mg/dL	1.1 (0.9–1.4]	1.1 (0.8–1.6)	0.400
Total bilirubin, mg/dL	2.5 (1.6–4.5)	2.4 (1.3–4.0)	0.132
CRP, mg/dL	4.7 (0.8–13.1)	8.1 (3.8–16.7)	< 0.001
SOFA score (median, IQR)	3.0 (2.0–4.0)	3.0 (2.0–5.0)	< 0.001
qSOFA score (N, %)			< 0.001
0	425 (76.4)	104 (55.0)	
1	108 (19.4)	64 (33.9)	
2	19 (3.4)	20 (10.6)	
3	4 (0.7)	1 (0.5)	
PMI, mm^2^/height m^2^ (median, IQR)	486.1 (380.8–599.1)	259.2 (218.9–346.2)	< 0.001
Treatment (N, %)			< 0.001
Antibiotics only	104 (18.7)	39 (20.6)	
Cholecystectomy	93 (16.7)	7 (3.7)	
ERCP combined	52 (9.4)	21 (11.1)	
ERCP only	234 (42.1)	78 (41.3)	
Percutaneous drainage	73 (13.1)	44 (23.3)	
ICU admission (N, %)	62 (11.2)	50 (26.5)	< 0.001
LOS, day (median, IQR)	8 (6–11)	10 (7–14)	< 0.001
Mortality on 14-day (N, %)	3 (0.6)	6 (3.4)	0.015
In-hospital Mortality (N, %)	4 (0.7)	11 (5.8)	< 0.001
Inotropic agents in ER (N, %)	26 (4.7)	22 (11.6)	0.001
Intubation in ER (N, %)	2 (0.4)	8 (4.2)	< 0.001

BMI, body mass index; COPD, chronic obstructive pulmonary disease; CKD, chronic kidney disease; CVA, cerebral vascular accident; SBP, systolic blood pressure; DBP, mean blood pressure; MAP, mean arterial pressure; HR, heart rate; RR, respiratory rate; BT, body temperature; WBC, white blood cell; BUN, blood urea nitrogen; CRP, C-reactive protein; SOFA, sequential organ failure assessment; PMI, psoas muscle index; ICU, intensive care unit; LOS, length of hospital stay.

There were significant differences in ICU admission, LOS, in-hospital mortality, 14-day mortality, inotropic agent use in the ER, and intubation in the ER between the sarcopenic and non-sarcopenic groups. The sarcopenic group had significantly higher rates of ICU admissions (26.5 vs. 11.2%), LOS (8 vs. 10 day), use of inotropic agents (11.6 vs. 4.7%), emergency intubation (4.2 vs. 0.4%), in-hospital mortality (5.8 vs. 0.7%), and 14-day mortality (3.4 vs. 0.6%).

**Figure 4:**
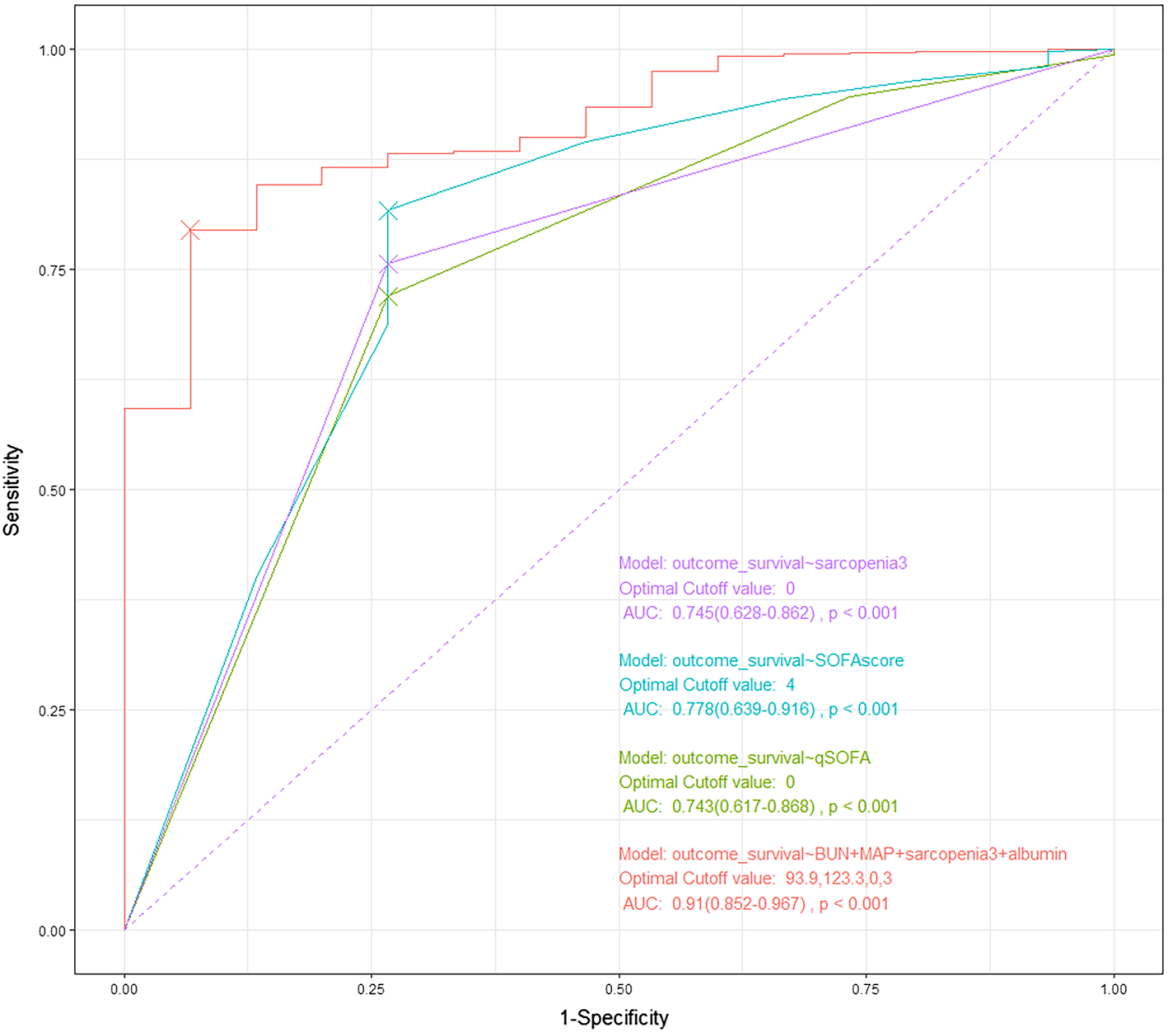
ROC curves of models for predicting in-hospital mortality of biliary sepsis. Blue line is a ROC curve of SOFA score, and AUC is 0.778. Green line is a ROC curve of qSOFA score, and AUC is 0.743. Purple line is a ROC curve of low muscle mass, and AUC is 0.745. Red line is a ROC curve of logistic regression model with variables including low muscle mass, BUN, albumin, and MAP (AUC 0.910).

Low muscle mass was a predictor of in-hospital mortality after biliary sepsis (Table 2) but was not a predictor of ICU admission. In multivariate logistic analysis, albumin, BUN, MAP, O2 saturation, low muscle mass, CRP, and Age were used as variables for ICU admission, and albumin, BUN, MAP, O2 saturation, low muscle mass, and Age were used as variables for in-hospital mobility. In the multivariate logistic model for predicting in-hospital mortality, albumin (OR, 0.34; 95% CI, 0.12–1.00), low muscle mass (OR, 3.81; 95% CI, 1.08–13.47), and BUN (OR, 1.03; 95% CI, 1.01–1.06) were independent prognostic indicators (Table 2). In the multivariate logistic model for predicting ICU admission, BUN (OR, 1.02; 95% CI, 1.00–1.03, albumin (OR, 0.40; 95% CI, 0.26–0.61), MAP (OR, 0.97; 95% CI, 0.95–0.98), and saturation (OR, 0.91; 95% CI, 0.86–0.97) were independent prognostic indicators (Table 2). In assessing the performance of our prediction models, the Brier score was calculated. The model for predicting ICU admissions achieved a Brier score of 0.098, indicating excellent performance level. Meanwhile, the model for predicting in-hospital mortality achieved a Brier score of 0.016, reflecting a good performance. In the logistic model, which predicted in-hospital mortality with MAP, BUN, and low muscle mass, albumin demonstrated the highest predictive power as a result of comparison with SOFA score and qSOFA score using the ROC curve (area under the curve of the logistic model vs. SOFA vs. qSOFA was 0.91 vs. 0.78 vs. 0.74) (Fig. 4).

**Table 2:** Multivariate logistic analysis with ICU admission and in-hospital mortality.

ICU admission	Multivariate analysis	In-hospital mortality	Multivariate analysis
Variables	OR (95% CI)	Variables	OR (95% CI)
Albumin	0.372 (0.240–0.578)	MAP	0.953 (0.918–0.989)
BUN	1.017 (1.001–1.032)	Albumin	0.339 (0.115–0.997)
MAP	0.962 (0.949–0.976)	BUN	1.031 (1.005–1.058)
O_2_ saturation	0.931 (0.877–0.989)	Low muscle mass	3.807 (1.076–13.467)
Low muscle mass	1.523 (0.905–2.563)	O_2_ saturation	0.984 (0.855–1.133)
CRP	1.002 (0.973–1.031)	Age	1.028 (0.965–1.095)
Age	1.008 (0.989–1.027)		

ICU, intensive care unit; BUN, blood urea nitrogen; CRP, C-reactive protein; MAP, mean arterial pressure.

In the subgroup analysis, the sarcopenic group had a significantly higher ICU admission rate than the non-sarcopenic group (30.8 vs. 13.5%, p = 0.03) and a significantly higher in-hospital mortality rate (12.8 vs. 1%, p = 0.01) in the antibiotic use group (conservative treatment group); however, this difference was not statistically significant. Invasive treatments (ERCP, percutaneous drainage, and surgery) had minimal impact on mortality. In the percutaneous drainage-only group, the ICU admission rate was higher in the non-sarcopenic group than in the sarcopenic group; however, there was no significant difference in mortality rates between the groups. There was no significant difference in in-hospital mortality according to the detailed treatment plan (percutaneous drainage vs. ERCP vs. surgery, or percutaneous drainage after ERCP surgery) (Table 3). These findings suggest that low muscle mass is associated with an increased likelihood of in-hospital mortality. However, invasive treatments mitigate this risk in patients with low muscle mass.

**Table 3:** Subgroup analysis comparing the prognosis of biliary sepsis treatment strategies between sarcopenic and non-sarcopenic groups.

	Non-Sarcopenic	Sarcopenic	P
Antibiotics only	104	39	
ICU admission (N, %)	14 (13.5)	12 (30.8)	0.032
In-hospital Mortality (N, %)	1 (1.0)	5 (12.8)	0.007
Percutaneous drainage	73	44	
ICU admission (N, %)	72 (98.6)	41 (93.2)	0.050
In-hospital Mortality (N, %)	1 (1.4)	3 (6.8)	0.296
ERCP only	234	78	
ICU admission (N, %)	19 (8.1)	13 (16.7)	0.052
In-hospital Mortality (N, %)	2 (0.9)	3 (3.8)	0.193
ERCP combined	52	21	
ICU admission (N, %)	4 (7.7)	4 (19.0)	0.321
In-hospital Mortality (N, %)	0 (0.0)	0 (0.0)	
Surgery	93	7	
ICU admission (N, %)	6 (6.5)	1 (14.3)	0.988
In-hospital Mortality (N, %)	0 (0.0)	0 (0.0)	

## Discussion

This study aimed to investigate the effect of low muscle mass on the prognosis of patients with biliary sepsis, including LOS, ICU admission, and in-hospital mortality. Our findings indicate that low muscle mass is a valuable predictor of these prognoses, particularly for predicting in-hospital mortality using logistic models, which demonstrated the highest predictive power. However, there was a very wide-ranged confidence interval in low muscle volume to predict in-hospital mortality. This may be influenced by the fact that low muscle mass is a binary factor, the incidence rate of in-hospital mortality was only 2.0%, and the ratio between the low muscle mass and non-low muscle mass groups was approximately 1:3.

### Association between low muscle mass and the prognosis of sepsis

Previous studies have reported similar results regarding the association between sarcopenia and the prognosis of sepsis. Oh et al. found a correlation between sarcopenia and short-term (28-days) and long-term (1 year and overall) mortality in 905 patients with septic shock^7^.

In addition, Cox et al. investigated the results of pre-existing muscle atrophy and acute muscle loss. Muscle mass changes were measured by analyzing CT scans of critically ill patients with intra-abdominal sepsis at 3-month and 12-month intervals. After diagnosing intra-abdominal sepsis in critically ill patients, muscle mass continued to decrease acutely. While there was a correlation between reduced quality of life and physical function for three months after sepsis, pre-existing muscle atrophy was independently associated with poor long-term functional status and increased one-year mortality^6^.

Okada et al. developed a "modified SOFA score" by adding sarcopenia to the SOFA score and evaluated its predictive performance. This study included 255 patients with various causes of sepsis admitted to the ICU and compared the 90-day mortality rates. This study revealed an association between psoas sarcopenia detected on CT and 90-day mortality^8^.

The relationship between sarcopenia and the prognosis of various diseases can be attributed to the central role of skeletal muscles in amino acid metabolism and immune responses under stressful conditions, such as sepsis^40^. In our study, the sarcopenia group had significantly lower serum albumin levels, which is consistent with the findings of a meta-analysis that reported lower serum albumin levels in patients with sarcopenia^41^. However, it remains unclear whether providing nutrients such as amino acids or protein supplements can improve disease prognosis.

### Association between treatment approaches and the prognosis of biliary sepsis

Kim et al. categorized patients diagnosed with acalculous cholecystitis (AAC) into surgical and non-surgical groups for comparison of outcomes. The surgical group comprised individuals undergoing open or laparoscopic cholecystectomy at AAC onset, while the non-surgical group included those opting for percutaneous transhepatic gallbladder drainage (PTGBD) with or without intravenous antibiotics. Although the average length of hospital stay was similar between both groups, the incidence of treatment-related complications significantly favored the non-surgical group (18.8 vs. 2.4%, p = 0.02). In the non-surgical group, there was no notable difference in recurrence rates between those receiving percutaneous drainage-only and antibiotic-only treatments (3.7 vs. 4.60%, p = 0.26), and no recurrence-related deaths were reported in either subgroup. The outcomes in the non-surgical group were as favorable as those in the surgical group. However, our subgroup analysis contrasted this, suggesting that invasive treatments counteracted the negative effects of low muscle mass. This discrepancy might arise from the distinct inclusion criteria of our study, which targeted severe cases with SOFA scores of 2 or higher and biliary sepsis, as opposed to Kim's study, which encompassed a broader range of conditions focusing solely on acute cholecystitis.

When examining mortality rates according to severity in the Tokyo Guidelines 13, it can be inferred that our study yielded different results because of the significant inclusion of mild cases in the population. The observed mortality rates were mild (Grade I) (1/161, 0.6%), moderate (Grade II) (0/60, 0%), and severe (Grade III) (3/14, 21.4%)^10^.

Another study investigated the association between mortality and antibiotic choice in ICU patients with severe acute cholangitis. This study found no significant differences in mortality rates between patients treated with amoxicillin and clavulanate and those treated with ceftriaxone and metronidazole. This study also compared different gallbladder decompression techniques (endoscopic, surgical, and percutaneous) and found no significant differences in mortality^42^. These findings are consistent with our grouping of patients who underwent invasive treatments for comparative purposes.

### Strengths

Our study stands out in its focus on sepsis prognosis from specific infection sources rather than studying sepsis as a whole with diverse causes. By emphasizing low muscle mass and biliary tract-related sepsis, we highlighted important insights. Additionally, our subgroup analysis revealed differences in ICU admissions and in-hospital mortality between sarcopenic and nonsarcopenic groups treated solely with antibiotics. However, aggressive treatments like percutaneous drainage, ERCP, or surgery showed no significant mortality differences based on muscle mass. In biliary sepsis, low muscle mass influenced mortality and ICU admission in conservative antibiotic treatment but didn't significantly impact outcomes in aggressive treatments. This suggests that invasive treatments may counter the adverse effects of low muscle mass. Lastly, our study minimized selection bias due to routine abdominal CT imaging for most biliary sepsis patients visiting the hospital.

### Limitations

However, this study has some limitations. First, the diagnosis of low muscle mass was based solely on the investigation of skeletal muscle mass at the third lumbar vertebra using CT. Physical performance and muscle strength assessments were not conducted because of the retrospective nature of the study. Second, it was difficult to determine a significant relationship between mortality and prognosis because the cohort had a low mortality rate of 6.5% (15/745). In a study by Bauer, the 30-day septic shock mortality rate was 33.7% (95% CI, 31.5–35.9) in North America, 32.5% (95% CI, 31.7–33.3) in Europe, and 26.4% (95% CI, 18.1–34.6) in Australia^9^. However, the Tokyo Guidelines suggest a mortality rate of approximately 2.7–10% for acute cholecystitis and cholangitis and approximately 1% for severe grade III disease^10^. The mortality rate for biliary sepsis, including cholangitis and cholecystitis in our study, was approximately 3.7–11.0%, which included a rate of 6.5% in our study^12^. Third, the results of the study can vary depending on the cut-off value used to define low muscle mass, and a consensus on the cut-off value for low muscle mass needs to be established. Statistics on low muscle mass in Asians are insufficient, and further studies are required to establish low muscle mass indices for various nationalities and races to investigate the relationship between low muscle mass and disease prognosis. Fourth, only SOFA and qSOFA scores were used to assess the severity of biliary sepsis. However, the lactate level, APACHE II score, and SAPS II score can also be used to determine the severity of sepsis. As we were limited by the retrospective nature of our study, we could not collect such data and apply these scores. Fifth, ICU admission, which was one of the outcomes of our study, was subjectively determined by emergency physicians and gastroenterologists because there were no clear criteria for ICU admission at our hospital. Sixth, the treatment plan for biliary sepsis was subjectively determined by the clinicians, which may have influenced the patient's prognosis. Seventh, subgroup analysis has few patients, this can lead to bias. Finally, our study had a small sample size and was a single-center study. Further studies are needed to resolve these limitations and provide strong evidence.

## Conclusion

Low muscle mass was significantly associated with in-hospital mortality and length of hospitalization in patients with biliary sepsis. Based on these results, earlier and more effective treatments should be considered for patients with low muscle mass and biliary sepsis. Further studies are warranted to overcome the limitations of this study and provide robust evidence in this field.

## Data Availability

The datasets used and/or analysed during the current study available from the corresponding author on reasonable request.

## References

[1] Cruz-Jentoft AJ (2019). Sarcopenia: Revised European consensus on definition and diagnosis. Age Ageing.

[2] Rolland Y, Van Kan GA, Gillette-Guyonnet S, Vellas B (2011). Cachexia versus sarcopenia. Curr. Opin. Clin. Nutr. Metab. Care.

[3] Ryan E (2019). Sarcopenia and inflammatory bowel disease: A systematic review. Inflamm. Bowel Dis..

[4] Welch C, Hassan-Smith ZK, Greig CA, Lord JM, Jackson TA (2018). Acute sarcopenia secondary to hospitalization: An emerging condition affecting older adults. Aging Dis..

[5] Montero-Errasquín B, Cruz-Jentoft AJ (2023). Acute sarcopenia. Gerontology.

[6] Cox MC (2021). The impact of sarcopenia and acute muscle mass loss on long-term outcomes in critically ill patients with intra-abdominal sepsis. J. Cachexia Sarcopenia Muscle.

[7] Oh HJ (2022). The impact of sarcopenia on short-term and long-term mortality in patients with septic shock. J. Cachexia Sarcopenia Muscle.

[8] Okada Y (2021). Predictive value of sarcopenic findings in the psoas muscle on CT imaging among patients with sepsis. Am. J. Emerg. Med..

[9] Bauer M (2020). Mortality in sepsis and septic shock in Europe, North America and Australia between 2009 and 2019—Results from a systematic review and meta-analysis. Crit. Care.

[10] Kimura Y (2013). TG13 current terminology, etiology, and epidemiology of acute cholangitis and cholecystitis. J. Hepato-Biliary-Pancreat. Sci..

[11] Rose Berlin Piodena-Aportadera M (2022). Calf circumference measurement protocols for sarcopenia screening: Differences in agreement, convergent validity and diagnostic performance. Ann. Geriatr. Med. Res..

[12] Hamaguchi Y (2016). Proposal for new diagnostic criteria for low skeletal muscle mass based on computed tomography imaging in Asian adults. Nutrition.

[13] Shen W (2004). Total body skeletal muscle and adipose tissue volumes: Estimation from a single abdominal cross-sectional image. J. Appl. Physiol..

[14] Tagliafico AS, Bignotti B, Torri L, Rossi F (2022). Sarcopenia: How to measure, when and why. La radiologia medica.

[15] Schlapbach, L. J. *et al.* Vol. 319 L518-L522 (American Physiological Society, Bethesda, 2020). 10.1152/ajplung.00369.2020 32812788

[16] Dellinger RP (2003). Cardiovascular management of septic shock. Crit. Care Med..

[17] Linde-Zwirble WT, Angus DC (2004). Severe sepsis epidemiology: Sampling, selection, and society. Crit. Care.

[18] Dombrovskiy VY, Martin AA, Sunderram J, Paz HL (2007). Rapid increase in hospitalization and mortality rates for severe sepsis in the United States: A trend analysis from 1993 to 2003. Crit. Care Med..

[19] Rhodes A, Evans L, Alhazzani W (2013). Surviving sepsis campaign: International guidelines for management of severe sepsis and septic shock. Crit. Care Med..

[20] Seymour CW (2017). Time to treatment and mortality during mandated emergency care for sepsis. N. Engl. J. Med..

[21] Singer M (2016). The third international consensus definitions for sepsis and septic shock (Sepsis-3). Jama.

[22] Nishida O (2018). The Japanese clinical practice guidelines for management of sepsis and septic shock 2016 (J-SSCG 2016). J. Intensive Care.

[23] Abe T (2018). Characteristics, management, and in-hospital mortality among patients with severe sepsis in intensive care units in Japan: the FORECAST study. Crit. Care.

[24] Heyland D (2015). The very elderly admitted to ICU: A quality finish?. Crit. Care Med..

[25] Mijnarends DM (2015). Instruments to assess sarcopenia and physical frailty in older people living in a community (care) setting: Similarities and discrepancies. J. Am. Med. Dir. Assoc..

[26] Caraballo C (2019). Association between site of infection and in-hospital mortality in patients with sepsis admitted to emergency departments of tertiary hospitals in Medellin, Colombia. Rev. Bras. Ter. Intensiva.

[27] Kim HB, Chun SY, Kim GW, Lim H, Cho YS (2023). Can sarcopenia predict poor prognosis of sepsis due to acute cholecystitis?. Am. J. Emerg. Med..

[28] De Paolis P (2002). Management of sepsis of the biliary tract: Indications to surgical treatment. Minerva Gastroenterol. Dietol..

[29] Kawaguchi Y (2019). Sarcopenia predicts poor postoperative outcome in elderly patients with lung cancer. Gen. Thorac. Cardiovasc. Surg..

[30] Kim G, Kang SH, Kim MY, Baik SK (2017). Prognostic value of sarcopenia in patients with liver cirrhosis: A systematic review and meta-analysis. PLoS ONE.

[31] Prashanthi PL, Ramachandran R, Adhilakshmi A, Radhan P, Sai V (2020). Standardization of PSOAS muscle index measurements using computed tomography. Int. J. Contemp. Med. Surg. Radiol..

[32] Ritz A (2021). Total psoas muscle area as a marker for sarcopenia is related to outcome in children with neuroblastoma. Front. Surg..

[33] Amini N (2015). Impact total psoas volume on short- and long-term outcomes in patients undergoing curative resection for pancreatic adenocarcinoma: A new tool to assess sarcopenia. J. Gastrointest. Surg..

[34] Fearon K (2011). Definition and classification of cancer cachexia: An international consensus. Lancet Oncol..

[35] Ganapathi AM (2014). Frailty and risk in proximal aortic surgery. J. Thorac. Cardiovasc. Surg..

[36] Mok M (2016). Prognostic value of fat mass and skeletal muscle mass determined by computed tomography in patients who underwent transcatheter aortic valve implantation. Am J. Cardiol..

[37] Okamura H (2019). The impact of preoperative sarcopenia, defined based on psoas muscle area, on long-term outcomes of heart valve surgery. J. Thorac. Cardiovasc. Surg..

[38] Paknikar R (2016). Psoas muscle size as a frailty measure for open and transcatheter aortic valve replacement. J. Thorac. Cardiovasc. Surg..

[39] Peng P (2012). Impact of sarcopenia on outcomes following resection of pancreatic adenocarcinoma. J. Gastrointest. Surg..

[40] Toshima T (2015). Profile of plasma amino acids values as a predictor of sepsis in patients following living donor liver transplantation: Special reference to sarcopenia and postoperative early nutrition. Hepatol. Res..

[41] Silva-Fhon JR, Rojas-Huayta VM, Aparco-Balboa JP, Cespedes-Panduro B, Partezani-Rodrigues RA (2021). Sarcopenia and blood albumin: A systematic review with meta-analysis. Biomedica.

[42] Touzani, S., El Bouazzaoui, A., Bouyarmane, F., Faraj, K., Houari, N., Boukatta, B., Kanjaa, N. Factors associated with mortality in severe acute cholangitis in a Moroccan intensive care unit: A retrospective analysis of 140 cases. *Gastroenterol. Res. Pract.* **2021**, 4583493. 10.1155/2021/4583493 (2021). 10.1155/2021/4583493 PMC7861946 33574838

